# Guttiferone K impedes cell cycle re-entry of quiescent prostate cancer cells via stabilization of FBXW7 and subsequent c-MYC degradation

**DOI:** 10.1038/cddis.2016.123

**Published:** 2016-06-02

**Authors:** Z Xi, M Yao, Y Li, C Xie, J Holst, T Liu, S Cai, Y Lao, H Tan, H-X Xu, Q Dong

**Affiliations:** 1 School of Pharmacy, Shanghai University of Traditional Chinese Medicine, Shanghai, China; 2 Engineering Research Center of Shanghai Colleges for TCM New Drug Discovery, Shanghai, China; 3 Discipline of Endocrinology, Royal Prince Alfred Hospital and Charles Perkins Centre, Sydney Medical School, The University of Sydney, Sydney, NSW, Australia; 4 School of Science and Health, The University of Western Sydney, Penrith South, Sydney, NSW, Australia; 5 Origins of Cancer Program, Centenary Institute, Camperdown, NSW, Australia; 6 Sydney Medical School, The University of Sydney, Sydney, NSW, Australia; 7 Children's Cancer Institute Australia for Medical Research, Sydney, NSW, Australia; 8 School of Women's and Children's Health, UNSW Medicine, Sydney, Australia

## Abstract

Cell cycle re-entry by quiescent cancer cells is an important mechanism for cancer progression. While high levels of c-MYC expression are sufficient for cell cycle re-entry, the modality to block c-MYC expression, and subsequent cell cycle re-entry, is limited. Using reversible quiescence rendered by serum withdrawal or contact inhibition in PTEN^null^/p53^WT^ (LNCaP) or PTEN^null^/p53^mut^ (PC-3) prostate cancer cells, we have identified a compound that is able to impede cell cycle re-entry through c-MYC. Guttiferone K (GUTK) blocked resumption of DNA synthesis and preserved the cell cycle phase characteristics of quiescent cells after release from the quiescence. In vehicle-treated cells, there was a rapid increase in c-MYC protein levels upon release from the quiescence. However, this increase was inhibited in the presence of GUTK with an associated acceleration in c-MYC protein degradation. The inhibitory effect of GUTK on cell cycle re-entry was significantly reduced in cells overexpressing c-MYC. The protein level of FBXW7, a subunit of E3 ubiquitin ligase responsible for degradation of c-MYC, was reduced upon the release from the quiescence. In contrast, GUTK stabilized FBXW7 protein levels during release from the quiescence. The critical role of FBXW7 was confirmed using siRNA knockdown, which impaired the inhibitory effect of GUTK on c-MYC protein levels and cell cycle re-entry. Administration of GUTK, either *in vitro* prior to transplantation or *in vivo*, suppressed the growth of quiescent prostate cancer cell xenografts. Furthermore, elevation of FBXW7 protein levels and reduction of c-MYC protein levels were found in the xenografts of GUTK-treated compared with vehicle-treated mice. Hence, we have identified a compound that is capable of impeding cell cycle re-entry by quiescent PTEN^null^/p53^WT^ and PTEN^null^/p53^mut^ prostate cancer cells likely by promoting c-MYC protein degradation through stabilization of FBXW7. Its usage as a clinical modality to prevent prostate cancer progression should be further evaluated.

Prostate cancer is remarkably heterogeneous ranging from the asymptomatic to symptomatic patients with widespread metastatic disease.^1^ Active surveillance has become an integral management option for selected cases of organ-confined prostate cancer.^2^ At present, 15% of patients with prostate cancer are monitored with active surveillance.^3^ While the morbidity of treatment may be avoided in a significant proportion of men on active surveillance, the progression of cancer is a major concern.^4^

The presence of quiescent cancer cells has been documented in many types of tumors.^5, 6^ These quiescent cancer cells are defined clinically as being Ki-67 negative, a protein marker of cells in cell cycle,^7, 8^ and reside in a reversible G_0_ arrest state.^9^ There is an increased percentage of Ki-67-negative prostate cancer cells in low-grade and low-volume prostate cancer compared with high-grade and high-volume cancers.^10^ This decrease in the fraction of Ki-67-negative cancer cells is correlated with disease severity^11^ and progression.^12^ These clinical observations suggest that an increase in the proportion of proliferative over quiescent cancer cells, or an increase in the transition from quiescence to a proliferative state, has an adverse impact on clinical outcome.

The levels of c-MYC expression are crucial for cell cycle re-entry. c-MYC mRNA levels are low in quiescent cells but increase within 1–3 h upon mitogen stimulation.^13^ Forced expression of c-MYC induces cell cycle re-entry of quiescent cells, but the downregulation or inactivation of c-MYC results in the impairment of cell cycle progression.^14^ Amplification of c-MYC is found in nearly half of human solid tumors,^15^ including 30% of prostate cancer.^14^

Owing to this critical role of c-MYC in cancer, understanding the mechanisms that regulate c-MYC protein expression may lead to new therapeutic opportunities. While c-MYC amplification is clearly important,^14, 15^ another mechanism of regulation is c-MYC protein degradation. ERK1/2, the effector of the RAS–RAF–MEK pathway, has been shown to stabilize c-MYC protein by phosphorylation at Ser62.^16^ Activation of AKT results in GSK3*β* phosphorylation, thereby inhibiting the normal function of GSK3*β* in destabilizing c-MYC via phosphorylation at Thr58.^16^ Hence, an increase in c-MYC protein stability can be expected when ERK1/2 and AKT are activated, which is common through gain-of-function mutations in RAS^17^ or loss-of-function mutations or deletion of PTEN^18^ in prostate cancer. Another mechanism of c-MYC regulation is through FBXW7 (F-box and WD repeat domain containing 7, E3 ubiquitin protein ligase), which plays a key role in c-MYC protein degradation in a Thr58-dependent manner,^19^ and this mechanism has been shown to play a critical role in leukemia-initiating cells.^20^

We have previously shown that Guttiferone K (GUTK), a bioactive polycyclic polyprenylated acylphloroglucinol, has the capability to induce cell cycle arrest at the G_0/_G_1_ phase in colon cancer cells.^21^ However, the mechanism of action, and whether GUTK can also impede cell cycle re-entry in quiescent cancer cells, has not been determined. In this present study, we describe for the first time that GUTK impedes cell cycle re-entry of quiescent PTEN^null^/p53^WT^ and PTEN^null^/p53^mut^ prostate cancer cells via stabilization of FBXW7 and subsequent c-MYC degradation.

## Results

### GUTK inhibits DNA synthesis after release from quiescence in prostate cancer cells

Experimental quiescence was achieved by serum withdrawal for 7 days in LNCaP cells (PTEN^null^/p53^WT^) or contact inhibition for 3 days in PC-3 cells (PTEN^null^/p53^mut^), and verified by propidium iodide (PI) analysis by flow cytometry and Ki-67 immunostaining (Supplementary Figures S1 and S2). These quiescent cancer cells were induced to re-enter cell cycle by either serum replenishment in LNCaP cells or re-plating of PC-3 cells at low density.

**Figure 1:**
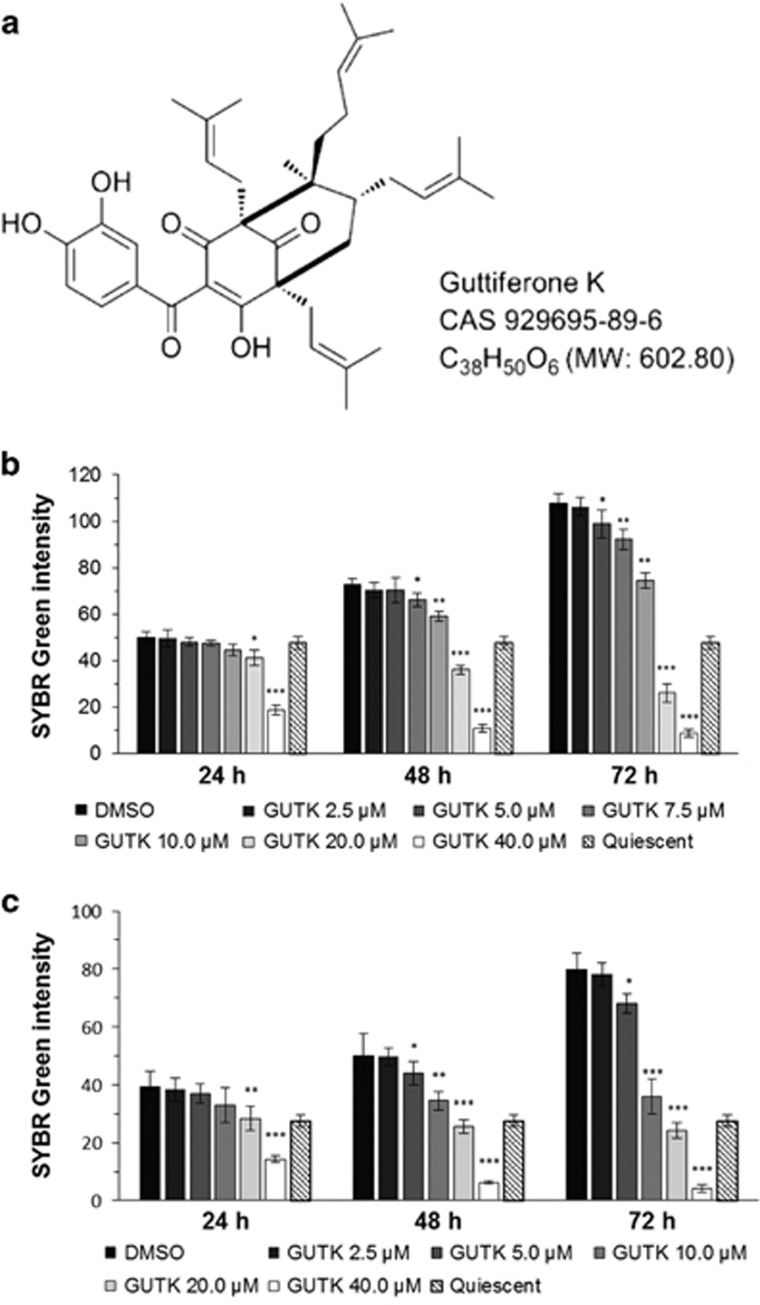
GUTK inhibited DNA synthesis following induction of cell cycle re-entry in prostate cancer cells. Chemical structure of GUTK (**a**). LNCaP (**b**) and PC-3 (**c**) cells was synchronized at quiescence by serum withdrawal for 7 days or contact inhibition for 3 days, before being induced to re-enter the cell cycle by serum restoration or re-plating at low density, respectively. GUTK at indicated concentrations was introduced at the time of induction and the cells were harvested and assessed for DNA content using the SYBR Green assay at 24, 48 and 72 h. Data are shown as the mean±S.D. of three independent experiments, *P<0.05, **P<0.01, ***P<0.001 *versus* control. Control cells (DMSO) were induced to re-enter the cell cycle in DMSO-containing medium without GUTK. Quiescent cells were analyzed to show DNA content prior to induction of cell cycle re-entry

The hallmark for cell cycle re-entry is the re-synthesis of DNA.^22^ We monitored the change in DNA content upon cell cycle re-entry in the presence or absence of Guttiferone K (GUTK; Figure 1a) with a SYBR Green assay. GUTK, introduced at the time when the cells were released from the quiescence, repressed the increase in DNA content seen in vehicle-treated control (dimethyl sulfoxide (DMSO)) in a dose- and time-dependent manner (Figures 1b and c). By comparing with the DNA content immediately before the induction for cell cycle re-entry (quiescence), GUTK was cytostatic at 2.5–10 *μ*M in both cell lines. At >20 *μ*M, the DNA content in both cell lines dropped below the baseline, suggesting GUTK at these concentrations was cytotoxic.

### GUTK delays cell cycle re-entry and division in prostate cancer cells

**Figure 2:**
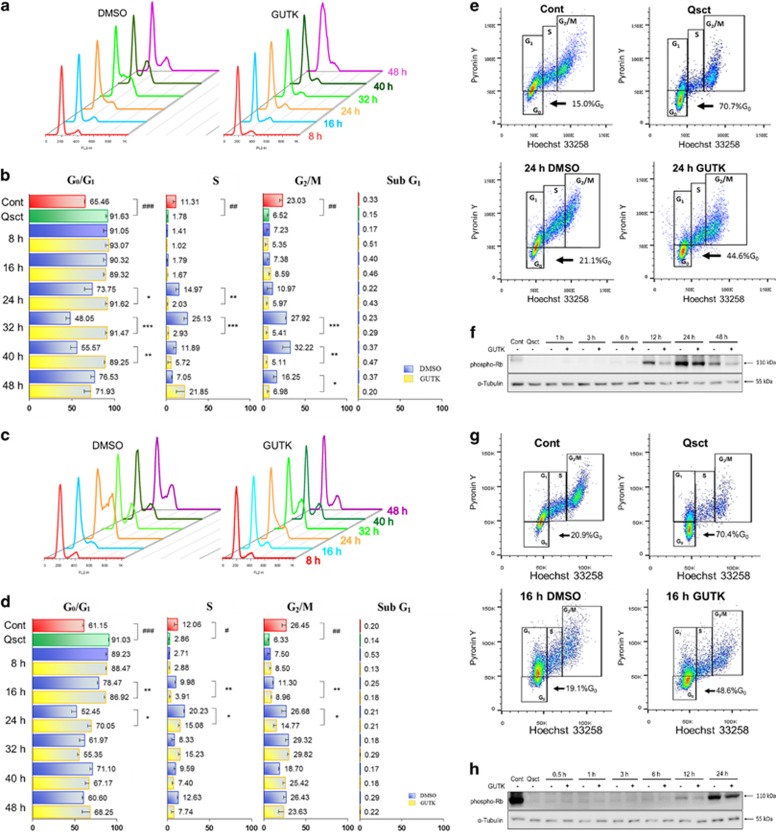
GUTK delayed cell cycle re-entry by quiescent prostate cancer cells. Quiescent LNCaP and PC-3 cells were induced to re-enter the cell cycle in the absence or presence of GUTK (GI_75_; Table 1). The cells were harvested at 8 h intervals following induction, fixed and kept at 4 °C prior to propidium iodide staining and flow cytometry. Representative flow cytometry images and quantification data of three independent experiments are shown for LNCaP (**a** and **b**) and PC-3 (**c** and **d**) cells. Cont (control cells: non-quiescent cells). Qsct (quiescent cells: LNCaP after serum withdrawal for 7 days or PC-3 after contact inhibition for 3 days). Data are expressed as the mean±S.D. of triplicate assays compared with non-quiescent controls (Control; ^#^*P*<0.05, ^##^*P*<0.01, ^###^*P*<0.001) or DMSO vehicle control cells at each time point (*P<0.05, **P<0.01, ***P<0.001). Flow cytometry of Hoechst 33258 and Pyronin Y double staining was used to detect G_0_ phase LNCaP (**e**) and PC-3 (**g**) cells in non-synchronized, synchronized, and during cell cycle re-entry with or without GUTK (GI_75_) at indicated times. Effects of GUTK (GI_75_) on phospho-Rb protein levels at indicated time points following induction of cell cycle re-entry in quiescent LNCaP (**f**) and PC-3 (**h**) cells was determined by immunoblotting. *α*-Tubulin served as a loading control

To examine the effects of GUTK on cell cycle progression, we first calculated the concentrations of GUTK at which the cytostatic action or growth inhibition (GI) reached 25% (GI_25_), 50% (GI_50_) and 75% (GI_75_) in LNCaP and PC-3 cells (Table 1). Next, quiescent LNCaP and PC-3 cells were induced to re-enter the cell cycle in the absence or presence of GUTK at GI_75_. The cells were harvested at 8 h intervals and subjected to PI staining and subsequent flow cytometric analysis. Upon release from quiescence, control LNCaP cells re-entered the cell cycle after approximately 24 h, as shown by a decreased proportion of cells in the G_0_/G_1_ phase, and increased the percentage of cells in the S and G_2_/M phases (Figures 2a and b). GUTK significantly delayed the re-entry of LNCaP cells at 24 h, with cell cycle re-entry occurring after approximately 48 h.

Control PC-3 cells re-entered the cell cycle more rapidly than LNCaP cells, after approximately 16 h following release from quiescence (Figures 2c and d). However, GUTK also delayed re-entry of PC-3 cells into cell cycle by approximately 8 h. It is noteworthy that GUTK at GI_75_ had no significant impact on cell viability based on sub-G_1_ fraction over these timeframes (Figures 2a–d).

Cells are diploid at G_0_ and G_1_ and thus it is not possible to differentiate them based on PI-stained DNA content. To enable separation of G_0_ and G_1_ populations, the cells were stained for DNA content with Hoechst 33258 and RNA content with Pyronin Y. Since G_0_ cells are ‘quiescent', they exhibit lower gene transcription, thereby resulting in low RNA content compared with G_1_ cells.^23, 24^ As expected, quiescence increased G_0_ cells in both LNCaP (Figure 2e) and PC-3 (Figure 2g) cells. During cell cycle re-entry, GUTK retained 24 and 30% more G_0_ cells than vehicle-treated control in LNCaP and PC-3 cells, respectively.

The phosphorylation state of the retinoblastoma protein (Rb) reflects cell cycle re-entry,^25^ with an increase in phosphorylated Rb (phospho-Rb) representing exit of cells from quiescence. The phospho-Rb protein at Ser807/811 was barely detectable until 12 and 24 h following cell cycle re-entry in control LNCaP and PC-3 cells, respectively (Figures 2f and h). However, GUTK-treated cells exhibited lower phospho-Rb at corresponding time points in both LNCaP and PC-3 cells. Taken together, these data suggest that GUTK is capable of impeding cell cycle re-entry by quiescent prostate cancer cells.

### Effects of GUTK on colony formation and tumorigenicity from quiescent prostate cancer cells

**Figure 3:**
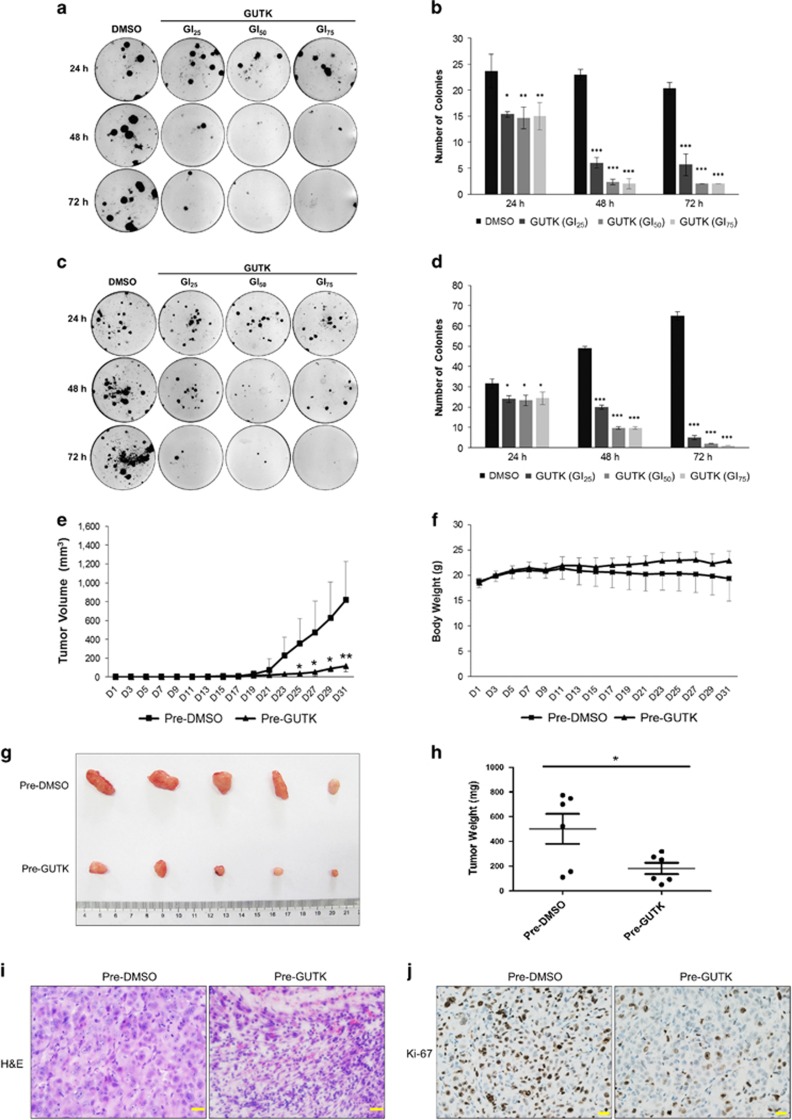
GUTK inhibited colony forming and tumorigenic capacity following induction of cell cycle re-entry. Quiescent LNCaP and PC-3 cells (50 cells/well) were induced to re-enter the cell cycle in the presence or absence of GUTK at GI_25_, GI_50_ and GI_75_ for 24, 48 and 72 h. Thereafter, the cells were cultured in the fresh full medium for additional 2 weeks. Emerging cell colonies were fixed, stained with crystal violet and imaged for counting. Representative image and quantification data of mean±S.D. of three independent experiments in LNCaP (**a** and **b**) and PC-3 (**c** and **d**). *P<0.05, **P<0.01, ***P<0.001 compared with the respective vehicle control. For *in vivo* analysis, 5-week-old male BALB/c nude mice were randomly divided into two groups (5 mice per group). Quiescent PC-3 cells were induced to re-enter the cell cycle by plating at a low density and treated either with GUTK at GI_75_ or with DMSO control for 72 h. The pre-treated cells were then subcutaneously injected into the left flank of male nude mice (day 1) and monitored for tumor formation. Tumor volume (**e**) and animal body weight (**f**) were measured every second day. At day 31, the tumors were resected, photographed (**g**) and weighed (**h**). The resected tumors were stained for H&E (**i**) and Ki-67 (**j**). Scale bar=20 *μ*m. All data are expressed as mean±S.D., *P<0.05, **P<0.01 compared with the DMSO pre-treated control

To determine the long-term effect of GUTK on the fraction of proliferative cells, LNCaP and PC-3 cells were treated with vehicle or GUTK at GI_25_, GI_50_ and GI_75_ for 24, 48 and 72 h from the release of quiescence. The cells were then cultured for an additional 14 days in the absence of GUTK at a density appropriate for formation of discernible colonies. Compared with the control, GUTK significantly decreased the number of colonies in a dose- and time-dependent manner (Figures 3a–d).

We then determined the tumorigenicity of cells after *in vitro* exposure to GUTK. PC-3 cells were induced to re-enter the cell cycle in the presence or absence of GUTK at GI_75_ for 72 h. The cells were then injected subcutaneously into nude mice. The cells treated with vehicle (DMSO) *in vitro* began to form measurable tumor at 21 days and continued their growth until termination at day 31 due to reaching ethical end point (Figure 3e). In contrast, the cells treated with GUTK *in vitro* exhibited delayed tumor formation, with measurable tumors only forming after 29–31 days. No significant change in animal body weight in the GUTK group was detected over the study period (Figure 3f). Analysis of tumors recovered at the end of study (day 31) showed that GUTK-treated cells developed smaller tumors (Figure 3g), which were significantly lighter compared with tumors formed from vehicle-treated cells (Figure 3h). Analysis of Ki-67 expression showed less positive cells in tumors formed from GUTK-treated cells compared with the vehicle group (Figures 3i and j). Taken together, these data further support the activity of GUTK in decreasing the proliferative fraction of prostate cancer cells.

### GUTK promotes c-MYC protein degradation by ubiquitin–proteasome pathway during cell cycle re-entry

**Figure 4:**
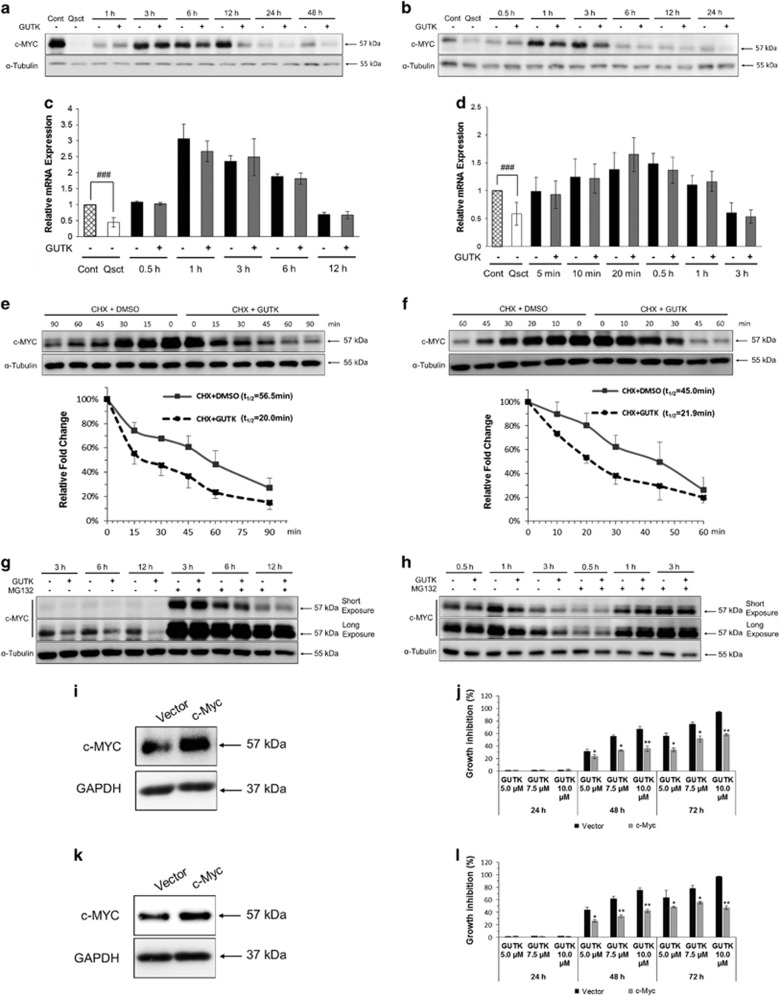
GUTK promoted the degradation of c-MYC via ubiquitin–proteasome pathway during cell cycle re-entry in prostate cancer cells. Effects of GUTK (GI_75_) on c-MYC protein levels at indicated time points following induction of cell cycle re-entry in quiescent LNCaP (**a**) and PC-3 (**b**) cells was determined by immunoblotting. *α*-Tubulin served as a loading control. The steady-state levels of c-MYC mRNA during cell cycle re-entry with or without GUTK at GI_75_ in LNCaP (**c**) and PC-3 (**d**) cells was examined by RT-qPCR. TBP was used for normalization. The data are expressed as the mean±S.D. of three independent experiments. ^#^*P*<0.001 compared with the indicated control. Quiescent LNCaP (**e**) and PC-3 (**f**) cells were induced to re-enter the cell cycle in the absence or presence of GUTK at GI_75_ for 3 and 1 h, respectively. The cells were subsequently exposed to 50 *μ*M cycloheximide (CHX) for indicated times, and the cell lysates were analyzed by immunoblotting. The plots were based on the mean value of three independent experiments, and the half-life (*t*_1/2_) of c-MYC protein was calculated by densitometric analysis using ImageJ software. Quiescent LNCaP (**g**) and PC-3 (**h**) cells were induced to re-enter the cell cycle in the absence or presence of GUTK at GI_75_ and at same time treated with 15 *μ*M MG132 or indicated times. The cell extracts were analyzed by immunoblotting. *α*-Tubulin served as a loading control. LNCaP cells after 5-day serum withdrawal and PC-3 cells after 1 day contact inhibition were transfected with a c-MYC expression vector or empty vector for 48 h. c-MYC protein levels in quiescent LNCaP (**i**) and PC-3 (**k**) cells were analyzed by immunoblotting. GAPDH served as a loading control. The effect of GUTK on DNA content in c-MYC-overexpressing LNCaP (**j**) and PC-3 (**l**) cells after release from quiescence for 24–72 h was determined by SYBR green assay. The growth inhibition was calculated using the equation described under SYBR Green assay in Materials and Methods. The data are expressed as the mean±S.D. of triplicate assays, *P<0.05, **P<0.01, compared with the corresponding negative control (empty vector)

To determine the mechanism by which GUTK impedes cell cycle re-entry of quiescent prostate cancer cells, we examined the protein levels of c-MYC in the presence or absence of GUTK at GI_75_. The c-MYC protein was barely detectable in quiescent LNCaP cells but reappeared 1–3 h after cell cycle re-entry (Figure 4a). However, the recovery of c-MYC protein level was repressed by GUTK 6 h onwards after cell cycle re-entry. In PC-3 cells, c-MYC protein levels increased after 0.5–1 h, with the inhibitory effect of GUTK on c-MYC protein levels appearing 1–3 h following induction of cell cycle re-entry (Figure 4b).

Since c-MYC mRNA levels were not altered by GUTK in either LNCaP (Figure 4c) or PC-3 cells (Figure 4d) over the same period, we examined the effect of GUTK on c-MYC protein stability. Quiescent LNCaP (Figure 4e) and PC-3 (Figure 4f) cells were induced to re-enter the cell cycle in the presence or absence of GUTK at GI_75_ for 3 and 1 h, respectively, before addition of cycloheximide (CHX; 50 *μ*M) to block protein translation. Samples were taken every 10–30 min to determine the decay rate of c-MYC protein. GUTK accelerated the decay rate of c-MYC, with the half-life (*t*_1/2_) of the c-MYC protein decreasing from 56.5 to 20.0 min in LNCaP, and 45.0 to 21.9 min in PC-3 cells. However, the negative effect of GUTK on c-MYC protein stability was abolished by treatment with 15 *μ*M proteasome inhibitor MG132 (Figures 4g and h), suggesting that the destabilizing effect of GUTK on c-MYC protein involves the ubiquitin–proteasome pathway. We also overexpressed c-MYC in quiescent LNCaP (Figure 4i) and PC-3 cells (Figure 4k), before inducing the cells to re-enter cell cycle in the presence or absence of GUTK. The suppressive effect of GUTK on DNA synthesis was significantly reduced in the cells overexpressing c-MYC (Figures 4j and l) compared with the empty vector-transfected cells. These data indicate that the inhibitory effect of GUTK on cell cycle re-entry involves promoting c-MYC protein degradation by the ubiquitin–proteasome pathway.

### GUTK reduces FBXW7 protein degradation during cell cycle re-entry of prostate cancer cells

**Figure 5:**
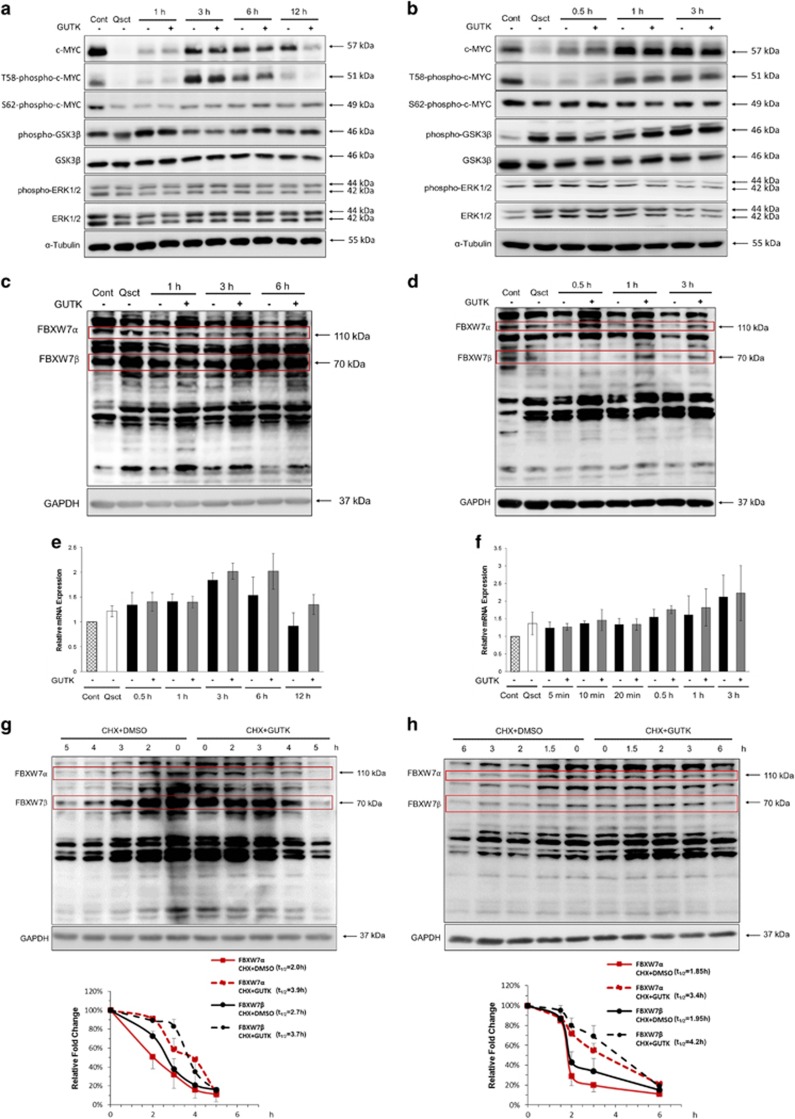
GUTK enhanced c-MYC protein degradation via increasing FBXW7 protein stability. Quiescent LNCaP (**a** and **c**) and PC-3 (**b** and **d**) cells were induced to re-enter the cell cycle in the absence or presence of GUTK at GI_75_ for indicated times. The total and phospho-c-MYC, total and phospho-GSK3*β*, total and phospho-ERK1/2, and FBXW7 *α* (110 kDa) and *β* (70 kDa) isoforms were analyzed by immunoblotting. The steady-state levels of FBXW7 mRNA in LNCaP (**e**) and PC-3 (**f**) cells during cell cycle re-entry with or without GUTK at GI_75_ were determined by RT-qPCR. The results are expressed as the mean±S.D. of triplicate assays. Quiescent LNCaP (**g**) and PC-3 (**h**) cells were induced to re-enter the cell cycle in the presence of 50 *μ*M cycloheximide (CHX) with or without GUTK at GI_75_ for indicated times and the cell lysates were analyzed by immunoblotting. The plots were based on the mean value of three independent experiments, and the half-life (*t*_1/2_) of FBXW7 *α* and *β* isoforms was calculated by densitometric analysis using ImageJ software. *α*-Tubulin or GAPDH served as a loading control

To determine the mechanism underlying the destabilizing effect of GUTK on c-MYC, we evaluated phospho-GSK3*β*, phospho-ERK1/2 and FBXW7 protein levels in quiescent LNCaP (Figure 5a) and PC-3 (Figure 5b) cells at cell cycle re-entry. The phosphorylation status of phospho-c-MYC at either Thr58 or Ser62 was not significantly altered by GUTK. Likewise, the two kinases responsible for the phosphorylation of c-MYC at Thr58 and Ser62, GSK3*β* and ERK1/2, did not change in either amount or phosphorylation due to GUTK.

**Figure 6:**
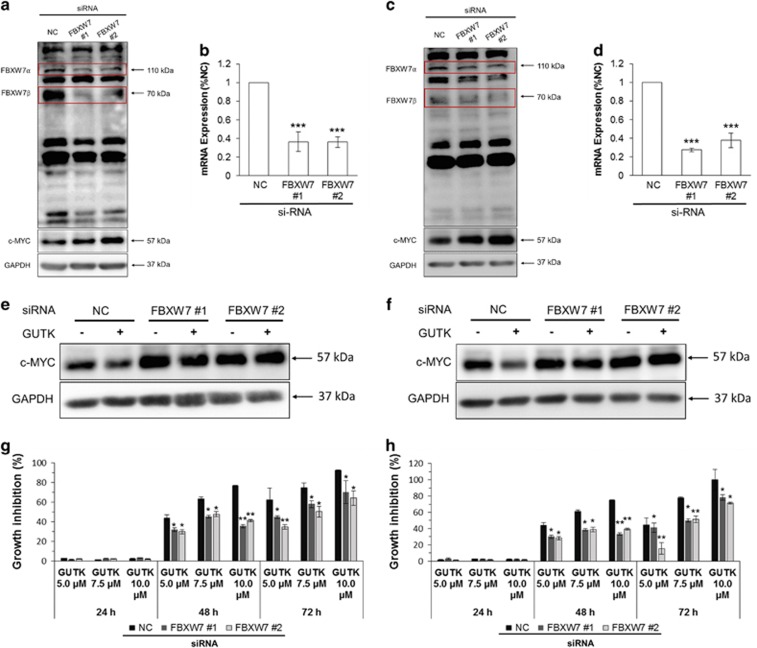
The effect of GUTK on cell cycle re-entry was diminished after FBXW7 knockdown. LNCaP cells following 5-day serum withdrawal and PC-3 cells at the day of contact inhibition were transfected with 50 nM siRNA duplexes targeting scramble or FBXW7 (#1 and #2) for 48 h (LNCaP) and 72 h (PC-3). FBXW7 *α* and *β* isoforms and c-MYC protein in quiescent LNCaP (**a**) and PC-3 (**c**) cells were analyzed by immunoblotting. GAPDH served as a loading control. The mRNA levels of FBXW7 were detected by RT-qPCR in quiescent LNCaP (**b**) and PC-3 (**d**) cells. TBP was used for normalization. After FBXW7 knockdown, c-MYC protein levels in LNCaP (**e**) and PC-3 (**f**) cells, which were induced for cell cycle re-entry with or without GUTK at GI_75_, were analyzed by immunoblotting. GAPDH served as a loading control. The effect of GUTK on DNA content in FBXW7-knockdown LNCaP (**g**) and PC-3 (**h**) cells after release from quiescence for 24–72 h was determined by SYBR green assay. The growth inhibition was calculated using the equation descried under SYBR Green assay in Materials and Methods. The data are expressed as the mean±S.D. of triplicate assays, *P<0.05, **P<0.01, ***P<0.001, compared with the corresponding negative control (NC)

FBXW7 has three isoforms, which, when individually expressed in HEK cells, show differential migration by western blot.^26^ While the *α* isoform is 79 kDa (NM_033632), it migrates on a western blot at 110 kDa by the widely used Bethyl antibody. The *β* and *γ* isoforms are 70 kDa (NM_018315) and 66 kDa (NM_001013415) respectively, and both migrate at the predicted size on western blot using the Bethyl antibody. We used the same antibody to verify the size of endogenous FBXW7 isoforms in FBXW7 siRNA-treated prostate cancer cell lines. We found that the 110 and 70 kDa bands were indeed reduced following the treatment with FBXW7 siRNA in the two prostate cancer cell lines (Figures 6a–d). However, the *γ* isoform was not clearly detected.

The levels of FBXW7 *α* and *β* isoforms were decreased following release from quiescence, but stabilized in the presence of GUTK in LNCaP (Figure 5c). The time course of this stabilization was between 3 and 6 h, which preceded the reduction in c-MYC in LNCaP cells. In PC-3 cells (Figure 5d), GUTK led to increased levels of FBXW7 *α* and *β* isoforms even after just 0.5 h, which also preceded its action on c-MYC. In contrast, another SCF complex E3 ubiquitin ligase, SKP2, which can also mediate c-MYC degradation,^27, 28^ did not exhibit altered protein levels until 24 h following the release from quiescence (Supplementary Figure S3).

As the steady-state levels of FBXW7 mRNA were comparable with or without GUTK in LNCaP (Figure 5e) and PC-3 (Figure 5f) cells, we determined the effect of GUTK on FBXW7 *α* and *β* isoform stability. Quiescent LNCaP (Figure 5g) and PC-3 (Figure 5h) cells were induced to re-enter cell cycle in the presence or absence of GUTK at GI_75_, together with 50 *μ*M CHX. The decay rate of FBXW7 *α* and *β* isoforms was substantially slower in the presence of GUTK.

To determine whether FBXW7 has a role in degrading c-MYC at quiescent state, we used siRNA to knock down FBXW7 expression in quiescent LNCaP (Figures 6a and b) and PC-3 (Figures 6c and d) cells. Both siRNAs suppressed the levels of FBXW7 *α* and *β* isoforms and FBXW7 mRNA, and led to an accumulation of c-MYC protein. To verify the role of FBXW7 in mediating GUTK action on c-MYC degradation and cell cycle re-entry, we treated both LNCaP and PC-3 cells with GUTK at induction of cell cycle re-entry in the presence or absence of FBXW7 siRNA. The suppressive effect of GUTK on c-MYC protein level and DNA synthesis was significantly reduced in cells transfected with the FBXW7 siRNA (Figures 6e–h). These data indicate that the effect of GUTK on inhibiting cell cycle re-entry is at least partly via stabilization of FBXW7 protein, thereby promoting c-MYC protein degradation.

### GUTK administered *in vivo* suppresses growth of quiescent prostate cancer cells

**Figure 7:**
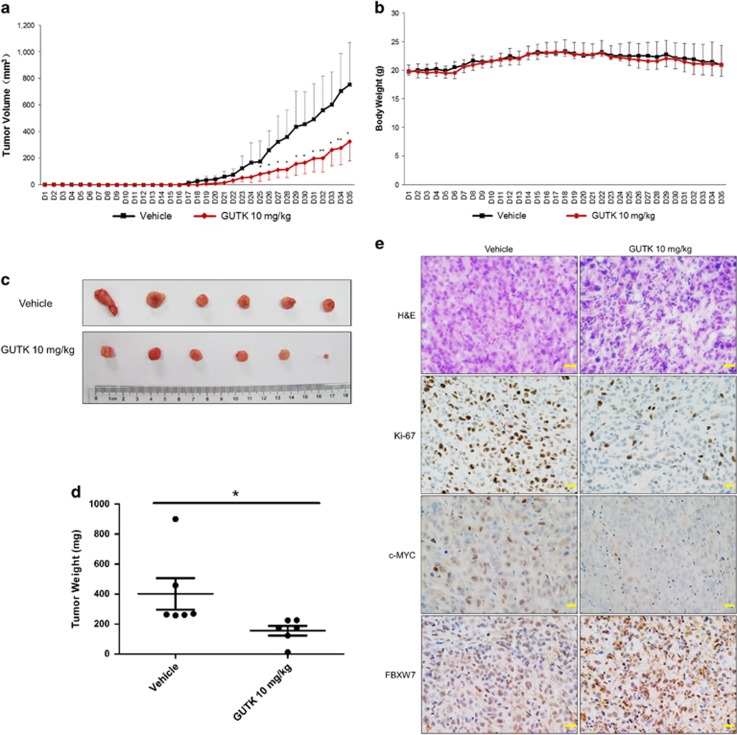
GUTK inhibits the growth of quiescent prostate cancer cells *in vivo*. Quiescent PC-3 cells were inoculated subcutaneously into the left flank of male nude mice. Mice received a daily intraperitoneal injection of GUTK (10 mg/kg), which begun one day prior to injection of the PC-3 cells (day 1) and continued for 34 days. Tumor volume (**a**) and body weight (**b**) of the animals were measured every day and expressed as mean±S.D. *P<0.05, **P<0.01 compared with the vehicle. At day 35, mice were killed and the tumors were resected, photographed (**c**) and weighed (**d**). Representative images of H&E staining and immunohistochemical staining for Ki-67, c-MYC and FBXW7 in tumor sections treated with the vehicle or GUTK (**e**). Scale bar=20 *μ*m

To determine whether *in vivo* administration of GUTK would be effective in inhibiting the progression of quiescent cancer cells into a tumor, GUTK or vehicle was injected intraperitoneally (10 mg/kg, daily) beginning at the day before subcutaneous implantation of quiescent PC-3 cells. There was an average reduction of 58% in tumor size and 61% in tumor weight at the end of experimental course (day 35) in the GUTK-treated group compared with the vehicle control (Figures 7a, c and d). No significant change in animal body weight was noted between the two groups (Figure 7b). Analysis of excised tumors showed a reduction in cell density (H&E), Ki-67 positivity and c-MYC protein levels, with an elevation of FBXW7 protein levels in GUTK-treated compared with the vehicle control (Figure 7e).

## Discussion

In an adult metazoan, most cells are in a quiescent state but are able to re-enter cell cycle to replace cells lost to injury or turnover. A transition from quiescence to proliferation is also an important mechanism utilized by cancer cells for self-renewing.^29^ While high levels of c-MYC expression are sufficient for cell cycle re-entry, the modality to block c-MYC expression is limited. In this study, we have identified a compound that is capable of impeding cell cycle re-entry by quiescent PTEN^null^/p53^WT^ and PTEN^null^/p53^mut^ prostate cancer cells, likely by promoting c-MYC protein degradation through stabilization of the E3 ubiquitin ligase FBXW7.

As expected, release from quiescence rapidly increased c-MYC protein levels. However, this increase was restrained in the presence of GUTK with an associated acceleration in c-MYC protein degradation. Further investigation on the decay of c-MYC protein confirmed an increased degradation through the ubiquitin–proteasome system. Both FBXW7 and SKP2 are E3 ubiquitin ligases from the F-box protein family that can participate in c-MYC degradation. FBXW7 has been shown to be a tumor suppressor in human carcinogenesis.^30^ The FBXW7 gene encodes three protein isoforms due to alternative splicing of mRNA.^31, 32, 33^ Previous studies have shown that cotransfection of each isoform with c-MYC could decrease c-MYC expression.^19^ Consistently, an inactivating mutation (R298H) in the first exon shared by all three isoforms diminished FBXW7-mediated c-MYC degradation.^19^ GUTK rapidly increased FBXW7 *α* and *β* isoform levels, preceding the decrease in c-MYC protein levels, suggesting that GUTK-induced decrease in c-MYC protein stability could be FBXW7-dependent. SKP2 was reduced at a time point hours after c-MYC degradation, and is therefore unlikely to be involved in this process. We propose that GUTK impedes cell cycle re-entry by stimulating c-MYC protein degradation through stabilization of FBXW7 protein, which is supported by facts that GUTK caused no significant change in FBXW7 mRNA levels, Thr58/Ser62-phospho-c-MYC and phospho-GSK3*β* and phospho-ERK1/2. Importantly, GUTK treatment also reduced c-MYC but increased in FBXW7 proteins in our xenograft model. Furthermore, the suppressive effect of GUTK on resumption of DNA synthesis upon cell cycle re-entry was diminished when FBXW7 expression was knocked down. It is worth noting that, although several proteins can stimulate c-MYC protein degradation, some can also stimulate c-MYC transcriptional activity.^34^ FBXW7 is one of the few that stimulate c-MYC degradation by ubiquitination^35^ and also inhibits c-MYC transcriptional activity.^34^

FBXW7 can also ubiquitinate a number of other oncogenes, including Notch1, cJUN and Cyclin E.^30^ Accordingly, FBXW7 is frequently mutated in a number of cancers, including colorectal carcinoma^36^ and T-ALL.^37^ While it has been reported previously that 5.6% of prostate cancer have mutation in FBXW7, this represented a single patient with a mutation in a cohort of 18 patients.^38^ We analyzed FBXW7 mutations across nine prostate cancer studies using cBioPortal (MSKCC), and showed only two mutations in FBXW7 in one study of 150 patients (1.3%), while there were no further mutations detected across more than 1000 patient samples in the other eight cohorts in this database. This suggests that there is a very low frequency of FBXW7 mutations in prostate cancer, and that protein expression may be more important than mutation in prostate cancer. How frequent the FBXW7 protein levels are reduced in prostate cancer is not clear at present. However, it has been shown that p53 mutation in prostatic small cell neuroendocrine carcinoma leads to a decrease in FBXW7 protein expression.^39^ Thus, developing new modalities to upregulate FBXW7 protein stability may be useful in combating a variety of cancers.

Induction of cell cycle re-entry following quiescence involves a rapid upregulation of critical transcription factors, signaling molecules and cell cycle proteins. As such, the activity of GUTK to inhibit this process relies on a potent effect on the master regulator c-MYC. GUTK also inhibited the growth of quiescent prostate cancer cells transplanted to nude mice. Importantly, we found no change in animal body weight following GUTK treatment. Based on gross anatomy and microscopic evaluation of heart, liver, spleen and kidney, we also found no noticeable change between GUTK-treated and vehicle control-treated mice. Since our first animal study using cancer cells that were pre-treated with GUTK *in vitro* showed a better efficacy than the second one (in which the treatment was conducted *in vivo*), further studies may be required in order to optimize GUTK treatment conditions or route of administration in mice.

Further study is needed to verify if GUTK can also exert a suppressive action on c-MYC independent of FBXW7. This could be achieved by overexpressing a FBXW7-resistant MYC mutant (T58A) in prostate cancer cells, and treating these cells with GUTK during cell cycle re-entry.

In conclusion, we have identified a natural compound from fruits that is able to impede cell cycle re-entry by quiescent prostate cancer cells. The mode of GUTK action is likely via stabilization of E3 ubiquitin ligase FBXW7 causing c-MYC ubiquitination and subsequent degradation. Since the two models of experimental quiescence were conducted in PTEN-null cancer cells with or without functional p53, the observed GUTK action in impeding cell cycle re-entry is likely to be maintained in PTEN-null cancer cells and be p53 status independent. Its usage as a clinical modality to prevent prostate cancer progression warrants further evaluation.

## Materials and Methods

### Chemicals and reagents

GUTK with a purity >98% was isolated from *Garcinia yunnanensis* Hu.^40^ The compound was dissolved in 100% DMSO and stored at −80°C. SYBR Green (S-7563), Trizol (15596-026), Lipofectamine 2 000 reagent (11668-019) and Lipofectamine RNAiMAX (13778-075) were obtained from Life Technologies (Carlsbad, CA, USA). RNase A (R6513), PI (P4170), cycloheximide (C7698), Hoechst 33258 (B2883), Pyronin Y (P9172) and DPX Mountant (317616) were from Sigma-Aldrich (Carlsbad, CA, USA). Protease inhibitor cocktail (11836145001) was from Roche Applied Science (Penzberg, Germany). DC protein assay kits (500-0113) were from Bio-Rad (Hercules, CA, USA). ECL Substrate kit (34078) was from Thermo Scientific (Carlsbad, CA, USA). PrimeScript RT Reagent Kit (RR037A) was from TAKARA Biotechnology (Japan). SYBR Green Realtime PCR Master Mix (QPK-201) was from TOYOBO Life Science (Osaka, Japan). Lysis buffer (P0013C) and Crystal Violet Staining Solution (C0121) were from Beyotime Institute of Biotechnology (Shanghai, China). MG132 (S2619) was from Selleck Chemicals (Shanghai, China). All cell culture supplies were from Life Technologies Gibco (Darmstadt, Germany).

### Cell lines and synchronization at quiescence

Human prostate cancer cell lines LNCaP and PC-3, obtained from the American Type Culture Collection (ATCC, Manassas, VA, USA), were cultured in RPMI 1640 medium supplemented with 10% FBS, penicillin at 100 units/ml and streptomycin at 100 *μ*g/ml (complete medium) at 37 °C in a humidified atmosphere with 5% _CO2_. LNCaP cell confluence was monitored until it reached 70–80% confluence. Thereafter, the serum-containing medium was replaced by serum-free medium for 7 days. PC-3 cell confluence was monitored until it reached 100% confluence. Thereafter, medium was changed and the confluence was maintained for 3 days. Cell cycle re-entry was rendered by serum replenishment for LNCaP and passage to a low density for PC-3 cells.

### SYBR Green assay

Quiescent LNCaP (10 000 cells/well) and PC-3 (7 000 cells/well) cells were seeded in 96-well plates and cultured with complete medium at various concentrations of GUTK. The same number of cells were kept as a baseline and stored at −80 °C. After treatment, the medium was aspirated and 100 *μ*l of lysis buffer containing SYBR Green at 1:10 000 dilution was added. The cells were lysed in the dark for 2 h. The frozen cells used as baselines were thawed, lysed in the same buffer and transferred to the same treatment plate. The fluorescence intensity of SYBR Green-stained DNA was measured using a plate reader (FLUOstar Omega, BMG Labtech, Germany) with excitation at 485/20 nm and emission at 528/20 nm. The net output of DNA was indicated by subtraction of total DNA (collected and measured at the end of experiment) by the input of DNA (i.e., baseline). The percentage of cell GI (GI_%_) was calculated by the equation: (net output of DNA in control−net output of DNA in treatment)/(net output of DNA in control) × 100%.

### Flow cytometric analysis

Quiescent LNCaP (2.5 × 10^5^ cells/well) and PC-3 (1.5 × 10^5^ cells/well) cells were seeded in a six-well plate and cultured with complete medium containing DMSO or GUTK. Cells were harvested every 8 h and fixed with ice-cold 70% ethanol and then stored at 4 °C overnight. PI flow cytometric analysis was performed as previously described.^41^ To distinguish G_0_ from G_1_ cells, the cells were incubated in 2 *μ*g/ml Hoechst 33258 in PBS at 37 °C for 45 min. Pyronin Y at 4 *μ*g/ml was directly added into each sample for a further 15 min incubation. DNA and RNA contents were determined using a flow cytometer (FACSCalibur II) equipped with CellQuest Pro software (BD Biosciences, San Jose, CA, USA), and analyzed using FlowJo software (version VX).

### Immunoblotting

After release from quiescence in the presence of DMSO or GUTK, cells were harvested and treated with ice-cold RIPA lysis buffer supplemented with a protease inhibitor cocktail. Protein quantification, electrophoresis and western blotting were performed as previously described.^42^ Antibodies (human specific) used were c-MYC (Abcam, Epitomics, Cambridge, UK, #1472-1), S62-phospho-c-MYC (ab51156), T58-phospho-c-MYC (ab28842) and GAPDH (ab128915) were obtained from Abcam Company (Cambridge, UK). Additional antibodies were phospho-Rb (Ser807/811) (#9308), ERK1/2 (137F5) (#4695), phospho-ERK1/2 (Thr202/Tyr204) (#4370), GSK3*β* (27C10) (#9315) and phospho-GSK3*β* (Ser 9) (#9323) purchased from Cell Signaling Technology (Danvers, MA, USA), FBXW7 (A301-721A) were obtained from Bethyl Laboratories (Montgomery, TX, USA) and *α*-tubulin (sc-5286) from Santa Cruz Biotechnology (Dallas, CA, USA).

Immunoblot images were exported in the format of tagged image file and quantified using ImageJ 1.46 software (National Institute of Health). After normalization to loading control, the immunoblot bands of c-MYC and FBXW7 were plotted to determine the half-life. The 50% decrease in protein intensity based on the *Y*-axis was used to determine the corresponding value on the *X*-axis, which by definition is the time required for the proteins of interest to decrease to the half of the baseline levels.

### Clonogenic assay

Quiescent LNCaP and PC-3 cells were seeded in six-well plates (50 cells/well) and incubated with complete medium containing various concentrations of GUTK. Cells were washed with PBS twice, before addition of complete media without DMSO or GUTK. Cells were cultured for 2 weeks to allow the formation of colonies, and the medium changed every 4 or 5 days. Cell colonies were fixed in 95% ethanol for 20 min and stained with 1% crystal violet in PBS. Each culture was photographed and the colonies consisting of more than 50 cells were scored.

### RT-qPCR

Quiescent LNCaP (2.5 × 10^5^ cells/well) and PC-3 (1.5 × 10^5^ cells/well) cells were seeded in a six-well plate and cultured with complete medium containing DMSO or GUTK. RNA was extracted by using Trizol reagent and 1 *μ*g of total RNA was reversed-transcribed with PrimeScript RT Reagent Kit. Quantitative PCR was performed with mRNA-specific primers in a StepOnePlus Real-Time PCR System (ABI) using the SYBR Green Realtime PCR Master Mix. Conditions for PCR were one cycle of 10 min at 95 °C; 40 cycles of 10 s at 95 °C and 30 s at 65 °C. The primer sequences were as follows: c-MYC forward, 5′-GCTGCCAAGAGGGTCA-3, and reverse, 5′-CGCACAAGAGTTCCGTAG-3′ FBXW7 forward, 5′-GCAACAGCAACTCAGACAACAA-3′ and reverse, 5′-GGAGTCCTCATCTACCGAAATAAAT-3′ TBP forward, 5′-GAACCACGGCACTGATTTTC-3′ and reverse, 5′-CCCCACCATGTTCTGAATCT-3′. Results were normalized to TBP, quantified using the manufacturer's software and analyzed with Microsoft Excel software.

### Transfection

LNCaP (6 × 10^5^ cells/well) cells were seeded in a six-well plate. After serum withdrawal for 5 days, cells were transfected with 50 nM siRNA targeting non-mammalian or FBXW7 sequence using Lipofectamine RNAiMAX for 48 h. Quiescent LNCaP cells were subsequently subjected to immunoblotting or SYBR Green assay. PC-3 (8 × 10^5^ cells/well) cells were seeded in a six-well plate and incubated to obtain 100% confluence. Then cells were transfected with 50 nM siRNA. After contact inhibition for 3 days, quiescent PC-3 cells were subjected to immunoblotting or SYBR Green assay. siRNAs for non-mammalian and FBXW7 were designed and synthesized by GenePharma (Shanghai Co., Ltd, Shanghai, China). Non-mammalian negative control siRNA, 5′-UUCUCCGAACGUGUCACGU-3′ and 3′-ACGUGACACGUUCGGAGAA-5′, FBXW7 #1 siRNA, 5′-GUCACGAACUCCAGUAGUATT-3′ and 3′-UACUACUGGAGUUCGUGACTT-5′, FBXW7 #2 siRNA, 5′-CUGAUGACAACACUUUAAATT-3′ and 3′-UUUAAAGUGUUGUCAUCAGTT-5′.

For overexpression of c-MYC, LNCaP (following 5-day serum withdrawal) and PC-3 cells (following 1 day contact inhibition) were transfected using Lipofectamine 2000 with either empty vector control (pcDNA3.1) or full-length c-MYC (Convenience Biology, Suzhou, China) in pcDNA3.1 for 4 h.

### Tumorigenicity of quiescent prostate cancer cells pre-treated with GUTK *in vitro*

Five-week-old male BALB/c nude mice were purchased from the Experimental Animal Center of the Chinese Academy of Science (Shanghai, China) and kept in a pathogen-free environment. Quiescent PC-3 cells were treated with DMSO or GUTK at GI_75_ for 72 h *in vitro*. Cells were trypsinized and washed twice with ice-cold PBS, then re-suspended in ice-cold 0.9% sodium chloride. The suspended cells were counted in the presence of 0.4% trypan blue and the concentration of viable cells was adjusted to 2 × 10^7^ cells per ml. Mice were randomly divided into two groups (5 mice per group), before 2 × 10^6^ cell pre-treated with either GUTK or DMSO cells were subcutaneously injected into the left flank of the animals. Tumor growth and body weight of the mice were monitored every other day. The tumor volume was assessed by measurement of its length (*L*) and width (*W*) using a digital caliper and calculated using the formula of (*L* × *W*^2^)/2. After 31 days, mice were humanely killed and the tumors were resected, photographed and weighed.

### Tumorigenicity of quiescent prostate cancer cells treated with GUTK *in vivo*

Five-week-old male BALB/c nude mice were randomly divided into GUTK treatment group and vehicle control group (6 mice per group). Daily intraperitoneal (i.p.) injection of vehicle (0.5% DMSO, 0.5% Tween 80) or GUTK at 10 mg/kg begun on the day before tumor implantation (day 1), and continued through to day 34. Quiescent PC-3 cells (2 × 10^6^ cells/mice) were subcutaneously injected into the left flank of the animals at day 2. Tumor volume and body weight of the mice were measured daily. After 35 days, mice were humanely killed and the tumors were resected, photographed and weighed.

### Immunohistochemistry

Tumors were resected immediately after euthanasia and subsequently fixed in 10% neutral-buffered paraformaldehyde at 4 °C for 48 h. The samples were embedded in paraffin, sectioned (5 *μ*m thickness) and stained with hematoxylin and eosin, Ki-67 (1:500; Abcam, ab92353), c-MYC (1:100) and FBXW7 (1:150). The color development was as previously described.^42^ The sections were mounted using DPX for histological analysis.

### Statistical analysis

The statistical software SPSS version 15.0 was used for statistical analysis. Student's two-tailed *t*-test was used for comparison between two different groups and ANOVA analysis was used with Fisher's LSD multiple-comparison test for multiple comparisons. All *P*-values <0.05 were considered statistically significant.

## Figures and Tables

**Table 1:** Growth inhibition (GI) values of GUTK in LNCaP and PC-3 cells during cell cycle re-entry

**GI_%_**	**LNCaP (*μ*M)**	**PC-3 (*μ*M)**
GI_25_	5.65±0.82	4.20±0.55
GI_50_	8.70±0.85	5.72±0.79
GI_75_	13.49±0.74	7.77±1.16

The concentrations of GUTK at which the GI reached 25% (GI_25_), 50% (GI_50_) and 75% (GI_75_) at 72 h from release from quiescence were determined with SYBR Green assay, using the 72 h data set (Figures 1b and c) by Probit function of SPSS version 15.0 software (IBM, Somers, NY, USA). GI values of each cell lines are shown as the mean±S.D. of three independent experiments

## Abbreviations

GUTK: Guttiferone K
c-MYC: v-myc avian myelocytomatosis viral oncogene homolog
FBXW7: F-box and WD repeat domain containing 7
PTEN: phosphatase and tensin homolog
p53: tumor suppressor protein p53
ERK: extracellular signal-regulated kinase
GSK3*β*: glycogen synthase kinase 3 beta
AKT: v-akt murine thymoma viral oncogene homolog
Rb: retinoblastoma protein
PI: propidium iodide
SKP2: S-phase kinase-associated protein 2
DMSO: dimethyl sulfoxide
H&E: hematoxylin–eosin staining
siRNA: small interfering RNA
CHX: cycloheximide
Cont: control cells
Qsct: quiescent cells
