# Supplementary material for: Guttiferone K impedes cell cycle re-entry of quiescent prostate cancer cells via stabilization of FBXW7 and subsequent c-MYC degradation
Source: Cell Death Dis. 2016 Jun 2;7(6):e2252–. doi: 10.1038/cddis.2016.123 (PMC5143372; doi:10.1038/cddis.2016.123)
Supplement: Supplementary Information [file cddis2016123x1.pdf]

SUPPLEMENTARY INFORMATION

Guttiferone K impedes cell cycle re-entry of quiescent prostate cancer cells *via* stabilization of FBXW7 and subsequent c-MYC degradation

Zhichao Xi, Mu Yao, Yang Li, Chanlu Xie, Jeff Holst, Tao Liu, Shuangfan Cai, Yuanzhi Lao, Hongsheng Tan, Hong-Xi Xu and Qihan Dong

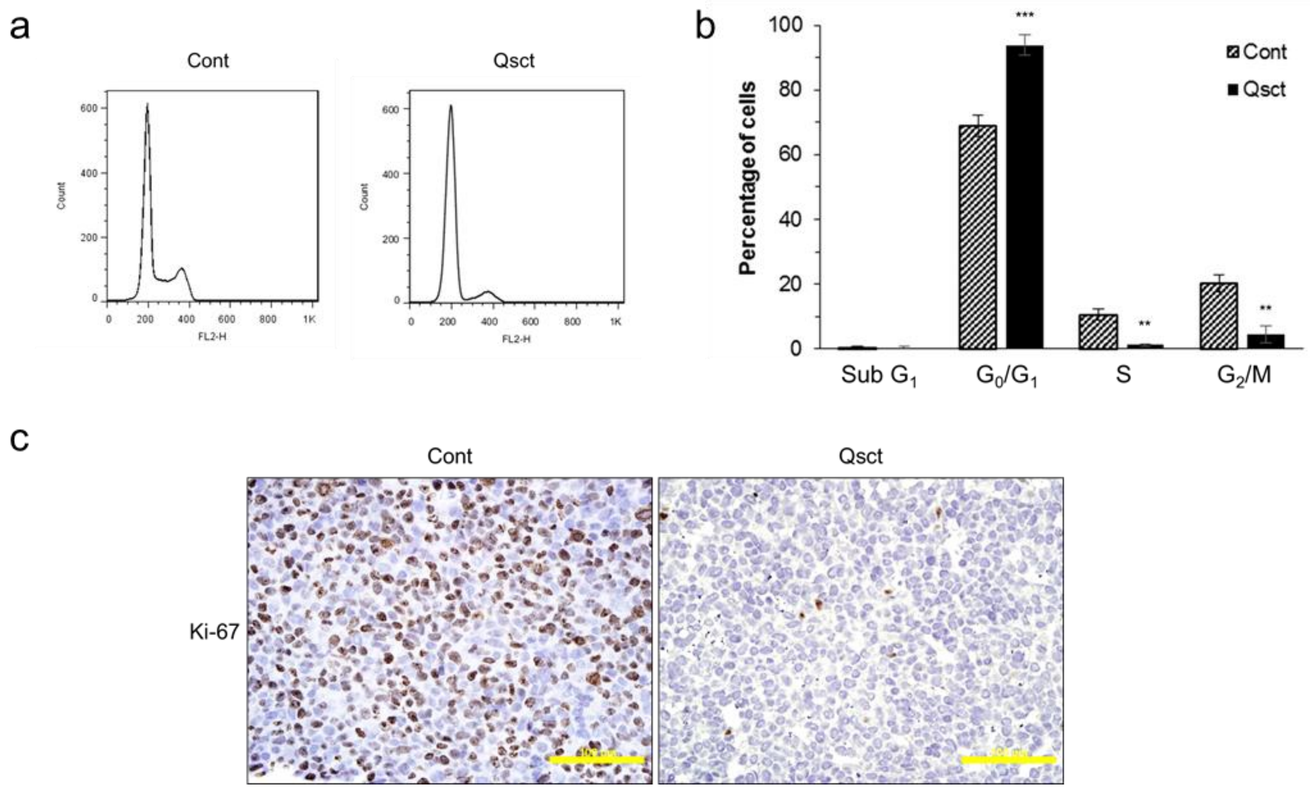

Supplementary Figure S1. Cell cycle phase of proliferative and quiescent LNCaP cells. LNCaP cells were rendered quiescent by serum withdrawal for 7 days, and compared to proliferating LNCaP cells by flow cytometric analysis of PI-stained cells (a, b) and Ki-67 immunostaining (c). Scale bar = 100 µm. Cont (control cells: non-quiescent cells). Qsct (quiescent cells: LNCaP after serum withdrawal for 7 days).

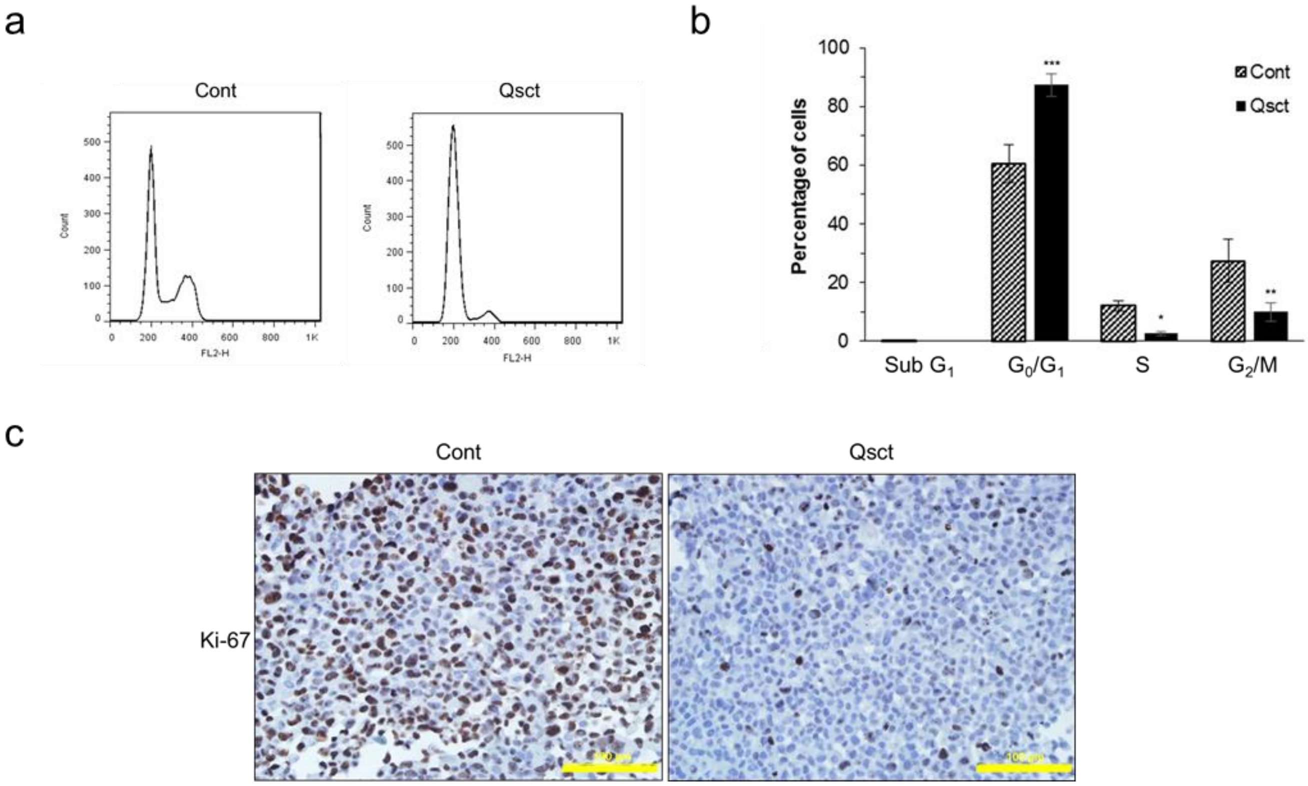

Supplementary Figure S2. The cell cycle phase of proliferative and quiescent PC-3 cells. PC-3 cells were rendered quiescent by contact inhibition for 3 days, and compared to proliferating PC-3 cells by flow cytometric analysis of PI-stained cells (a, b) and Ki-67 immunostaining (c). Scale bar = 100 μm. Cont (control cells: non-quiescent cells). Qsct (quiescent cells: PC-3 after contact inhibition for 3 days).

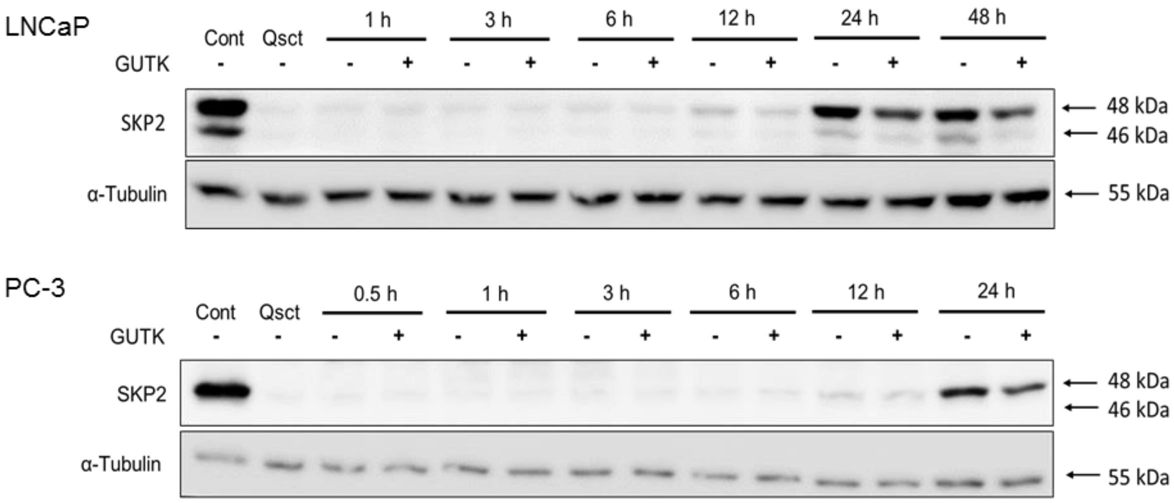

Supplementary Figure S3. Analysis of SKP2 protein levels after GUTK. Effect of GUTK (GI_{75}) on SKP2 protein levels by immunoblotting, after induction of cell cycle re-entry for indicated times. α-Tubulin served as a loading control. **Related to Figure 5.**
